# Genetic Mapping of the Incompatibility Locus in Olive and Development of a Linked Sequence-Tagged Site Marker

**DOI:** 10.3389/fpls.2019.01760

**Published:** 2020-01-28

**Authors:** Roberto Mariotti, Alice Fornasiero, Soraya Mousavi, Nicolò G.M. Cultrera, Federico Brizioli, Saverio Pandolfi, Valentina Passeri, Martina Rossi, Gabriele Magris, Simone Scalabrin, Davide Scaglione, Gabriele Di Gaspero, Pierre Saumitou-Laprade, Philippe Vernet, Fiammetta Alagna, Michele Morgante, Luciana Baldoni

**Affiliations:** ^1^ CNR - Institute of Biosciences and Bioresources (IBBR), Perugia, Italy; ^2^ Institute of Applied Genomics, Udine, Italy; ^3^ Department of Agricultural, Food, Environmental and Animal Sciences, University of Udine, Udine, Italy; ^4^ IGA Technology Services, Udine, Italy; ^5^ University of Lille, CNRS, UMR 8198 - Evo-Eco-Paleo, F-59000, Lille, France; ^6^ ENEA - Trisaia Research Centre, Rotondella, Italy

**Keywords:** genetic map, *Olea europaea*, double digest restriction associated deoxyribonucleic acid sequencing, self-incompatibility, functional markers

## Abstract

The genetic control of self-incompatibility (SI) has been recently disclosed in olive. Inter-varietal crossing confirmed the presence of only two incompatibility groups (G1 and G2), suggesting a simple Mendelian inheritance of the trait. A double digest restriction associated DNA (ddRAD) sequencing of a biparental population segregating for incompatibility groups has been performed and high-density linkage maps were constructed in order to map the SI locus and identify gene candidates and linked markers. The progeny consisted of a full-sib family of 229 individuals derived from the cross ‘Leccino’ (G1) × ‘Dolce Agogia’ (G2) varieties, segregating 1:1 (G1:G2), in accordance with a diallelic self-incompatibility (DSI) model. A total of 16,743 single nucleotide polymorphisms was identified, 7,006 in the female parent ‘Leccino’ and 9,737 in the male parent ‘Dolce Agogia.’ Each parental map consisted of 23 linkage groups and showed an unusual large size (5,680 cM in ‘Leccino’ and 3,538 cM in ‘Dolce Agogia’). Recombination was decreased across all linkage groups in pollen mother cells of ‘Dolce Agogia,’ the parent with higher heterozygosity, compared to megaspore mother cells of ‘Leccino,’ in a context of a species that showed exceptionally high recombination rates. A subset of 109 adult plants was assigned to either incompatibility group by a stigma test and the diallelic self-incompatibility (DSI) locus was mapped to an interval of 5.4 cM on linkage group 18. This region spanned a size of approximately 300 Kb in the olive genome assembly. We developed a sequence-tagged site marker in the DSI locus and identified five haplotypes in 57 cultivars with known incompatibility group assignment. A combination of two single-nucleotide polymorphisms (SNPs) was sufficient to predict G1 or G2 phenotypes in olive cultivars, enabling early marker-assisted selection of compatible genotypes and allowing for a rapid screening of inter-compatibility among cultivars in order to guarantee effective fertilization and increase olive production. The construction of high-density linkage maps has led to the development of the first functional marker in olive and provided positional candidate genes in the SI locus.

## Introduction

In cultivated olive (*Olea europaea* subsp. *europaea* var. *europaea*), the cross breeding activities have been delayed by the particularly long generation time (Santos-Antunes et al., 2005), the extended juvenile phase, the high demanding nursery practices, such as the forcing of seedling growth (Rugini et al., 2016) and the time and space needed for plant growing (Picheny et al., 2017). In olive, breeding programs last about 30 years on average (Lavee et al., 2014; Rallo et al., 2016) and have been limited to the empirical selection of a few sporadic intraspecific crosses (Rallo et al., 2008), or to clonal selection (Manaï et al., 2007; Gomes et al., 2008; Trapero et al., 2013; Mousavi et al., 2019), while the timing for the selection of new cultivars in other fruit crops has been greatly reduced, also by the application of new efficient genomic tools (Biscarini et al., 2017; Laurens et al., 2018; Cai et al., 2019). However, the importance of olive cultivation at worldwide level and the new challenges posed by the ongoing climate change, are leading to an ever increasing demand for new cultivars (Gutierrez et al., 2009; Urban, 2015; Bosso et al., 2016).

One of the current limitations to olive productivity is represented by its complex self- and inter-incompatibility system (Saumitou-Laprade et al., 2017a; Alagna et al., 2019), a barrier that may seriously curb yield and restrict the varietal choice for planting to only a few inter-compatible or self-fertile varieties. Fruit set deficiencies due to ineffective pollination are generally underestimated by the farmers, however, it has been demonstrated that supplemental pollination may significantly increase olive production (Ayerza and Coates, 2004), indicating the importance of an effective pollination design of olive orchards.

In olive as well as in other species of the Oleaceae family, such as *Phillyrea* (*Phillyrea angustifolia*) and ash (*Fraxinus excelsior*), an incompatibility system known as diallelic self‐incompatibility (DSI) has been described (Saumitou-Laprade et al., 2010; Vernet et al., 2016; Saumitou-Laprade et al., 2018). It has been hypothesized that two alleles at the DSI locus exist in cultivated olives, S and s, with S dominant over s, with only two possible genotypic combinations (Ss and ss), corresponding to G1 and G2 incompatibility groups, respectively, and by stigma test analysis it was never found G1xG1 or G2xG2 compatibility (Saumitou-Laprade et al., 2017a), where G1xG2 crosses always generate G1:G2 = 1:1 balanced progenies. All olive cultivars seem to be self-incompatible, even if pseudo-self-fertility might occur for some cultivars in particular conditions (Alagna et al., 2019).

Although the DSI mechanism in olive is known, many important aspects remain to be clarified, such as the location of the incompatibility locus on the genome, the identification of candidate genes controlling this trait, and markers closely linked to incompatibility. The availability of such information will allow for a systematic screening of olive cultivars to identify their group of incompatibility through genotyping with linked markers.

Olive is a diploid species (2n = 2x = 46) with a genome size of approximately 1.4 Gb (Cruz et al., 2016), with a mean C-value of 1.59 pg (1.56 Gb), where more than 30% sequences are represented by tandem repeats (Barghini et al., 2014). Up to now, only intraspecific crosses have been used for olive mapping, and very early studies were performed with dominant markers (de la Rosa et al., 2003; Wu et al., 2004; Aabidine et al., 2010; Khadari et al., 2010). Recently, more dense maps have been produced by the use of codominant markers, such as diversity arrays technology (DArT) (Domínguez-García et al., 2012; Atienza et al., 2014), simple-sequence repeat (SSR) (Sadok et al., 2013), and single-nucleotide polymorphism (SNP) markers (Marchese et al., 2016; İpek et al., 2017; Unver et al., 2017).

SNPs are sequence-tagged markers widely used for association and genetic mapping due to their wide distribution along the genome, high-throughput genotyping, and ease to score (Vezzulli et al., 2008; Deulvot et al., 2010; Lou et al., 2017). Molecular markers linked to the traits of interest can be identified through different strategies, such as genetic linkage mapping based on biparental populations (Curtolo et al., 2017; Ji et al., 2018; Zheng et al., 2018; Sapkota et al., 2019), or through genome-wide association studies (GWAS), based on unrelated individuals (Khan et al., 2013; Nicolas et al., 2016; Elsadr et al., 2019). The generation of high-resolution linkage maps, a prerequisite for gene positional cloning, allows the genetic dissection of quantitative trait loci, assists in comparisons of synteny, and provides marker order for anchoring sequence scaffolds or physical contigs to linkage groups. In perennial fruit crops, the availability of markers tightly linked to traits under selection may strongly facilitate the progress of breeding programs (Minamikawa et al., 2018).

Linkage analysis can be performed on multi-generation families derived by the cross, back-cross, or selfing of homozygous or heterozygous lines. In fruit trees, the use of these progenies is hindered by the lack, in most cases, of homozygous genotypes and by the long generation time (Bai et al., 2007; Jaillon et al., 2007). For this reason, full-sib F1 families deriving from inter- or intra-specific varietal crosses of highly heterozygous parents are generally used in tree species (Bartholomé et al., 2015) and linkage analysis is conducted separately for each parent using a two-way pseudo-testcross mapping strategy (Grattapaglia and Sederoff, 1994). Pseudo-testcross mapping has been carried out in many fruit crops, such as apricot (Irisarri et al., 2019), peach (Zeballos et al., 2016), oil palm (Bai et al., 2018), clementine (Ollitrault et al., 2012), grapevine (Zhu et al., 2018), apple (Di Pierro et al., 2016), and forest trees, like poplar (Zhigunov et al., 2017) and oak (Bodénès et al., 2016). A number of markers closely linked to important simple or complex traits has been identified with this strategy (Zhu et al., 2015; Zhai et al., 2016). Marker-trait associations, either for qualitative or quantitative trait loci (QTL), should allow to predict in advance the final breeding value of genotypes, permitting to discard unwanted genotypes immediately after seed germination and to only grow individuals that will later display the traits of interest (Edge-Garza et al., 2015; Migicovsky and Myles, 2017).

In the present work, a F1 progeny derived from the cross of two highly heterozygous and completely self-incompatible cultivars, ‘Leccino’ × ‘Dolce Agogia,’ respectively belonging to G1 and G2 groups of incompatibility (Saumitou-Laprade et al., 2017b), was genotyped by ddRAD sequencing technology in order to construct linkage maps. This crossing was performed because parents show different phenotypes for numerous important agronomical traits. In particular, ‘Dolce Agogia’ is a vigorous cultivar, with an alternate bearing, resistant to some of the most dangerous olive diseases, such as *Verticillium dahliae* and *Spilocaea oleagina* (Iannotta and Scalercio, 2012; Arias-Calderón et al., 2015), whereas ‘Leccino’ has medium vigor and constant bearing, is susceptible or only partially resistant to the pathogens indicated above (Salman, 2017), but it was recently reported as tolerant to *Xylella fastidiosa* (Giampetruzzi et al., 2016), the most devastating emerging plant pathogen for the Mediterranean agriculture (Suffert et al., 2009). The maps generated in the present work, represent a significant improvement over the previous ones (Marchese et al., 2016; İpek et al., 2017; Unver et al., 2017), because they include a higher number of SNP markers with a uniform genome distribution and can, therefore, serve as saturated maps for trait mapping. We used the ‘Leccino’ map to identify the location of the DSI locus, to select sequence scaffolds of ‘Leccino’ that were anchored to the DSI by linked markers, and to develop and validate SNP markers for the incompatibility trait.

## Materials and Methods

### Plant Material and Deoxyribonucleic Acid Extraction

A full-sib F1 progeny of 229 individuals was generated from the cross between ‘Leccino,’ as female parent, and ‘Dolce Agogia,’ as male parent. The progeny, thereafter referred to as Le×DA, is represented by a first set of 16-year-old seedlings (amounting to 155 individuals) and by a second set of 5 years old seedlings (74 individuals) grown in an experimental field, for a total of 229 F1 genotypes. The progeny showed high phenotypic variability, in particular for the length of the juvenile phase, plant vigor, tree habit, fruit bearing, and cutting’s rooting ability (Hedayati et al., 2015). DNA was extracted from leaves using the DNeasy Plant Mini Kit (Qiagen).

### Parentage Analysis of the Progeny

‘Leccino,’ ‘Dolce Agogia,’ and the Le×DA progeny were genotyped with a set of SSR markers for confirming the parentage (Baldoni et al., 2009). PCRs were performed as previously reported (Mousavi et al., 2017) and the amplified fragments were separated on an ABI 3130 Genetic Analyzer capillary sequencer (Applied Biosystems, Foster City, CA). Alleles were called using the GeneMapper 3.7 software (Applied Biosystems, Foster City, CA). Parentage analysis was performed using CERVUS version 3.0.7 program (Kalinowski et al., 2007) to sort out seedlings derived from open pollination.

### Phenotyping for Incompatibility Groups of the Le×DA Progeny

Those offspring that have reached the mature phase and started blooming (109 out of 229, all belonging to the first set of 16-year-old seedlings), were phenotyped for the incompatibility group. Data on 91 individuals were previously reported (Saumitou-Laprade et al., 2017a), all the others were *de novo* phenotyped following the same protocol previously applied. In order to minimize errors, phenotyping of all individuals was repeated on previously clonally propagated plants grown in a separate field. Before blooming, flowering twigs of each tree were protected from open pollination with blossom bags. At full blooming, bagged twigs were collected and 20 open flowers per tree were used for the analysis. Sepals and petals were removed from 10 flowers and pistils were placed in two separate plates (one for each of the two pollen donors) onto Brewbacker and Kwack medium. The stigmas of five pistils in one plate were pollinated with pollen of ‘Leccino’ (G1) and the other five with pollen of ‘Dolce Agogia’ (G2). From the remaining 10 flowers, pollen was collected and used for pollinating ‘Leccino’ and ‘Dolce Agogia’ stigmas. The same protocol applied by Saumitou-Laprade et al. (2017a) was used to observe the growth of pollen tubes on the stigmata.

### Double Digest Restriction Associated Deoxyribonucleic Acid Sequencing of Parents and Progeny

Genomic DNA was digested with *Sph*I and *Mbo*I restriction enzymes, following a double digest restriction associated DNA sequencing (ddRADseq), according to the method proposed by Peterson et al. (2012) and modified by Scaglione et al. (2015). Fragments were added to a ligation reaction containing barcoded adapters, pooled, and then fractioned by agarose gel-electrophoresis. DNA in the size range between 350 and 600 bp was purified using a QIAquick Gel Extraction kit (Qiagen, Venlo, Netherlands). Enrichment PCR was performed with PCR primers that incorporate Illumina hybridization/sequencing sites and index sequences for combinatorial multiplexing. Quality, quantity, and reproducibility of libraries were assessed using a Caliper instrument (DNA High Sensitivity chip). Sequencing was carried out on an Illumina HiSeq 2500 instrument, generating 125-bp paired-end reads.

### Single-Nucleotide Polymorphism Calling

Reads were aligned using Bowtie 2 software (Langmead and Salzberg, 2012) with default parameters against a whole-genome assembly of ‘Leccino’ (Muleo et al., 2016). The reference consisted of 509,032 scaffolds, with N50 length of 10,037 bp, amounting to a total of 1.429 giga base pairs (Gbp). Alignments with mapping quality < 4 were removed. Segregating sites were identified using the software Stacks (Catchen et al., 2011) and a bounded SNP model with alpha = 0.05 and upper error (epsilon) of 0.1. Genotypes were called with a minimum coverage of eight reads. Heterozygous genotypes were called within a read coverage ratio of 0.15-0.85 for reference and alternate alleles. Segregating loci were retained if genotypes were called in > 150 progeny.

### Genetic Mapping

Genetic maps were generated with the double pseudo-test-cross approach for each parent using SNP genotypic data and phenotypic binary data for the incompatibility group. Linkage groups were obtained using the R/qtl module of the R statistical package with a logarithm of odds (LOD) threshold > 10 and a recombination rate of 0.20. Markers were first ordered using MSTmap (Wu et al., 2008) with default parameters. A Perl implementation of the SMOOTH program (van Os et al., 2005) was used to remove errors within haplotypes. Markers were re-ordered using MSTmap. Marker distances were calculated using the Kosambi function {map distance in centi-Morgan (cM) equals ¼ ln [(1 + 2r)/(1 − 2r)], where r is the observed recombination frequency}. Markers with distorted segregation were identified using a χ^2^ test (α = 0.05).

### Comparative Genomic Analyses

Scaffolds of ‘Leccino’ that were anchored to the DSI locus by flanking markers were aligned with the genome assembly of the wild olive (*Olea europaea* var. *sylvestris*, GCA_002742605.1) using (B)LASTZ. Gene annotation was performed using the highest hit in blastp alignments with the NCBI protein database.

### Development of a Sequence-Tagged Site Marker for Diallelic Self-Incompatibility

PCR primers were designed on the sequence of scaffold_6030 using the program Primer3 version 4.0 (forward 5’-3’: TTTTGGGTGCGAATTGTCCA, reverse 5’-3’: AGGCCACTGTATTTCTAACTCG) for the amplification of a 476-bp fragment spanning two adjacent SNPs that co-segregate with DSI. PCRs were performed using 25 ng of template DNA and Q5 High-Fidelity DNA polymerase (New England Biolabs). The thermal profile consisted of 98°C for 30 s, followed by 35 cycles at 98°C for 10 s, 60°C for 20 s, and 72°C for 30 s, and a final extension at 72°C for 2 min. Amplicon size was checked by 1% agarose gel electrophoresis. Amplicons of the expected size showing unique bands were sequenced using the BigDye Terminator v1.1 Cycle Sequencing Kit (Thermo Fisher Scientific) and an ABI PRISM 3130 XL Genetic Analyzer (Applied Biosystems, Foster City, CA). The obtained sequences were aligned using BioEdit 7.1.7 (www.mbio.ncsu.edu/BioEdit/bioedit.html), to identify polymorphisms. In order to phase single-nucleotide substitutions in heterozygous individuals and to obtain full sequences from individuals carrying heterozygous small indels, amplicons were cloned by using pGEM-T Easy Vector (Promega) and *Escherichia coli* XL1 blue strain. DNA from different colonies for each genotype was amplified and sequenced as described above.

## Results

### Genetic Maps

The parentage analysis allowed to validate the origin of 95% of the 241 seedlings for a total of 229 true-to-type F1 genotypes. The remaining 12 individuals were excluded from further analyses. A total of 16,743 segregating loci were identified in the Le×DA progeny by ddRAD sequencing. The parental maps consisted of 23 linkage groups, including 9,737 RAD loci in ‘Dolce Agogia’ and 7,006 RAD loci in ‘Leccino’ (**Table 1**). Linkage groups were numbered consistently with the chromosome numbering of the wild olive genome assembly (Unver et al., 2017). The male parental map (‘Dolce Agogia’) included 1,829 genetic bins (i.e., positions on the genetic map with a unique segregation pattern) for a total length of 3,538 cM. The female parental map (‘Leccino’) included 2,311 genetic bins for a total length of 5,680 cM. The average distance between adjacent genetic bins was 2.46 cM in ‘Leccino’ and 1.93 cM in ‘Dolce Agogia’.

**Table 1:** Statistics of the parental linkage maps. Chromosome number (Chr), restriction associated DNA (RAD) markers, length (cM), and bins are reported for ‘Leccino’ and ‘Dolce Agogia’.

Chr	Leccino			Dolce Agogia		
	RAD markers	cM	Bins	RAD markers	cM	Bins
1	268	232.874	98	522	165.979	95
2	355	216.454	101	341	169.818	82
3	308	207.305	91	312	127.713	63
4	222	221.467	72	377	117.929	65
5	262	179.227	81	387	89.749	52
6	378	333.996	120	773	192.604	100
7	304	229.924	113	487	143.868	85
8	291	207.785	99	263	133.481	53
9	225	198.075	75	317	166.185	72
10	527	474.952	187	715	283.975	148
11	402	443.421	160	607	196.433	113
12	382	263.992	119	484	210.657	101
13	298	252.841	97	443	286.844	82
14	262	260.901	88	379	112.722	62
15	369	241.160	113	632	140.747	96
16	264	205.234	89	199	127.797	54
17	294	233.648	95	369	129.562	65
18	357	188.878	97	424	169.573	85
19	340	290.418	112	404	196.065	91
20	182	154.145	63	337	127.779	65
21	205	186.505	71	289	95.021	50
22	286	231.551	90	364	151.210	77
23	225	225.217	80	312	102.030	73
Total	7,006	5,679.970	2,311	9,737	3,537.741	1,829

All linkage groups of the ‘Leccino’ map were consistently longer that those of the ‘Dolce Agogia’ map, presumably as a result of a consistently higher recombination rate between homologous chromosomes in megaspore mother cells of ‘Leccino’ than in pollen mother cells of ‘Dolce Agogia.’ The shorter map obtained in ‘Dolce Agogia’ was not explained by runs of homozygosity at the chromosome *termini* compared to ‘Leccino,’ except for the upper 34 cM of Linkage Group (LG) 16 in ‘Leccino,’ which lacked segregating sites in ‘Dolce Agogia.’ The longer map obtained in ‘Leccino’ was, on the other hand, not explained by the presence of isolated markers with high likelihood of genotypic errors, which usually locally inflate genetic distances due to inconsistent genotype calls compared to neighboring markers. It was noteworthy that the suppression of recombination was observed in the parent with the higher level of heterozygosity, as revealed by the larger number of segregating sites.

### Structural Variation Between Parental Genomes and Segregation Distortion

**Figure 1:**
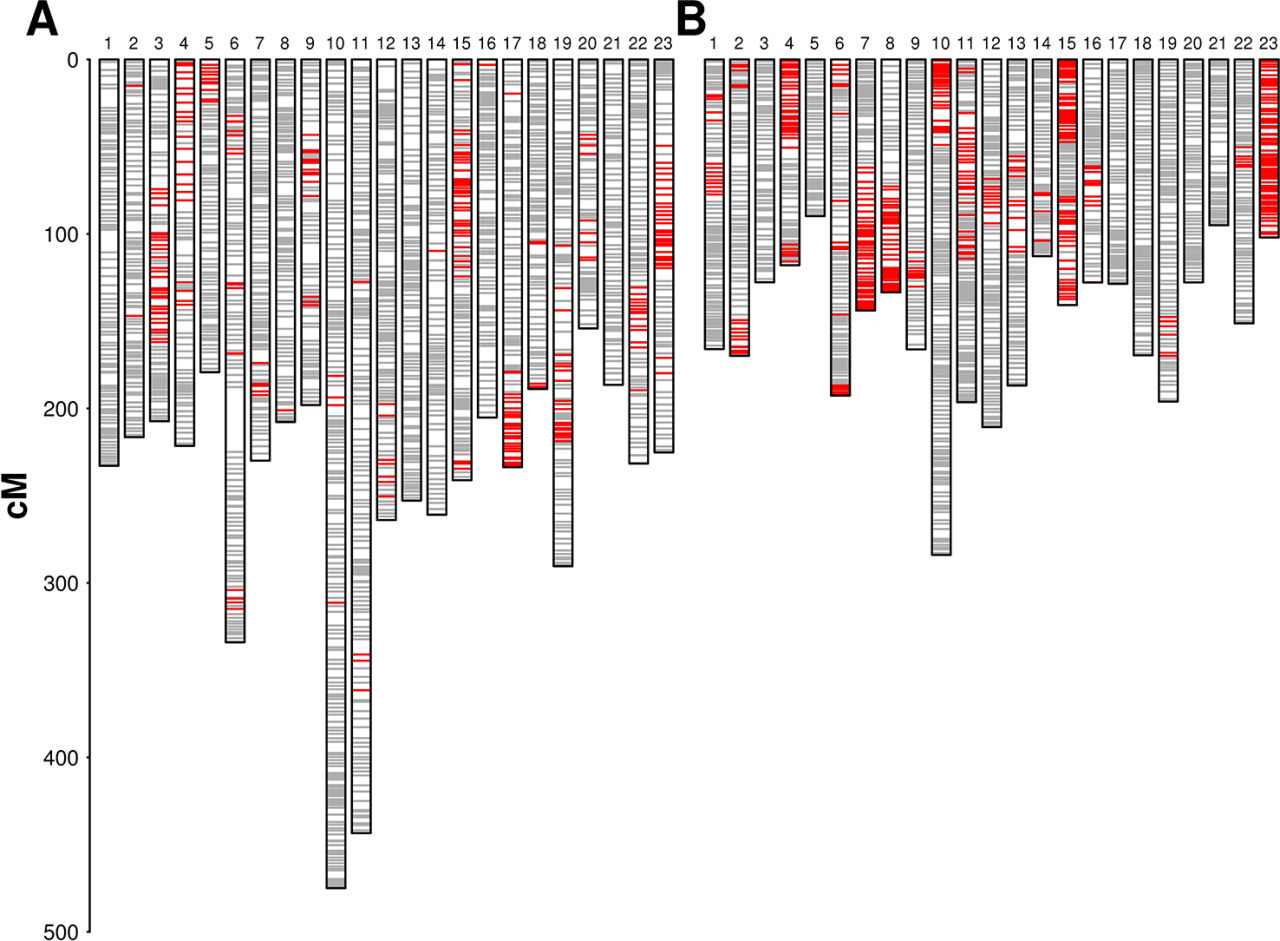
Parental genetic maps: diagrams show linkage groups in ‘Leccino’ (panel **A**) and ‘Dolce Agogia’ (panel **B**). Markers are plotted in gray. Markers with distorted segregation (α = 0.05) are plotted in red. LGs are not oriented with respect to the conventional orientation of chromosomes in the wild olive genome assembly.

We used 2,305 RAD loci segregating from both parents with an allelic status “ABxCD” for pairing and aligning linkage groups between parental maps. The alignment of the two parental maps did not show inter-chromosomal translocations. Two large inversions were detected in LG 1, involving a segment of about 30 cM, and in LG 8, involving a segment of about 12 cM. We did not detect suppression of recombination around each inversion in any of the parental maps and we mapped segregating sites within the inverted regions in each parental map. In both cases, the parental genomes were therefore homozygous for alternative structural variants, but the haplotypes carrying the inversion were ancient enough to have accumulated segregating sites. Minor rearrangements in marker order were identified on LGs 2, 4, 5, 7, and 17. Extended regions with distorted segregation (α = 0.05) were detected on several linkage groups in both maps, with a higher frequency being observed in ‘Dolce Agogia’ map (**Figure 1**). Localized distortion was also detected at markers flanking the lower side of the incompatibility locus (see below) with an excess of marker alleles in phase with the incompatibility recessive allele.

### Identification of the Incompatibility Locus

A stigma test was performed on 106 adult Le×DA individuals. The incompatibility group phenotyping assigned 56 individuals to the G1 group, 47 to the G2 group, and discarded 3 individuals due to uncertain phenotype. Segregation of the phenotype followed the expected 1:1 ratio (χ^2^ = 0.79). A self-pollination test of ‘Leccino’ and ‘Dolce Agogia’ showed no growth of pollen tubes on the stigmas, confirming their complete self-incompatibility.

**Figure 2:**
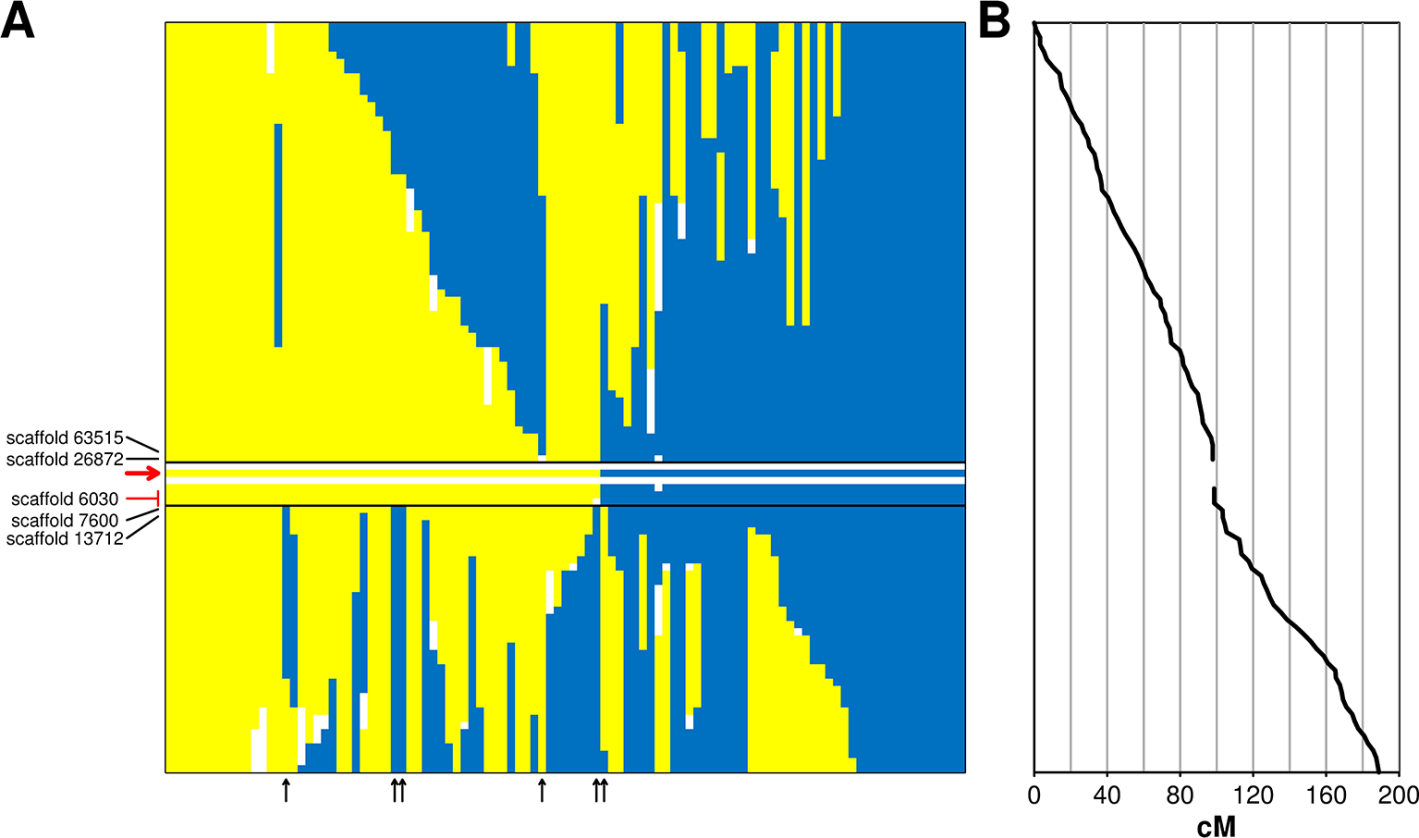
Panel **(A)**: genetic interval controlling the incompatibility system on linkage group 18 of cv. Leccino. The diallelic self-incompatibility (DSI) locus is delimited by black lines, the red arrow points to the DSI phenotype. Each vertical bar shows native and recombinant chromosomes of ‘Leccino’. The homolog carrying the S allele is shown in yellow, the homolog carrying the s allele is shown in blue and the same color indicate the G1 and G2 phenotypes, respectively. Blanks indicate missing genotypes. Flanking restriction associated DNA (RAD) markers and corresponding scaffolds are shown in black. Co-segregating RAD markers are shown in red and the corresponding scaffold is black. Vertical black arrows indicate the diagrams of recombinant chromosomes in the seedlings that defined the genetic borders of the DSI locus. Panel **(B)**: genetic distance from the top of LG 18.

**Figure 3:**
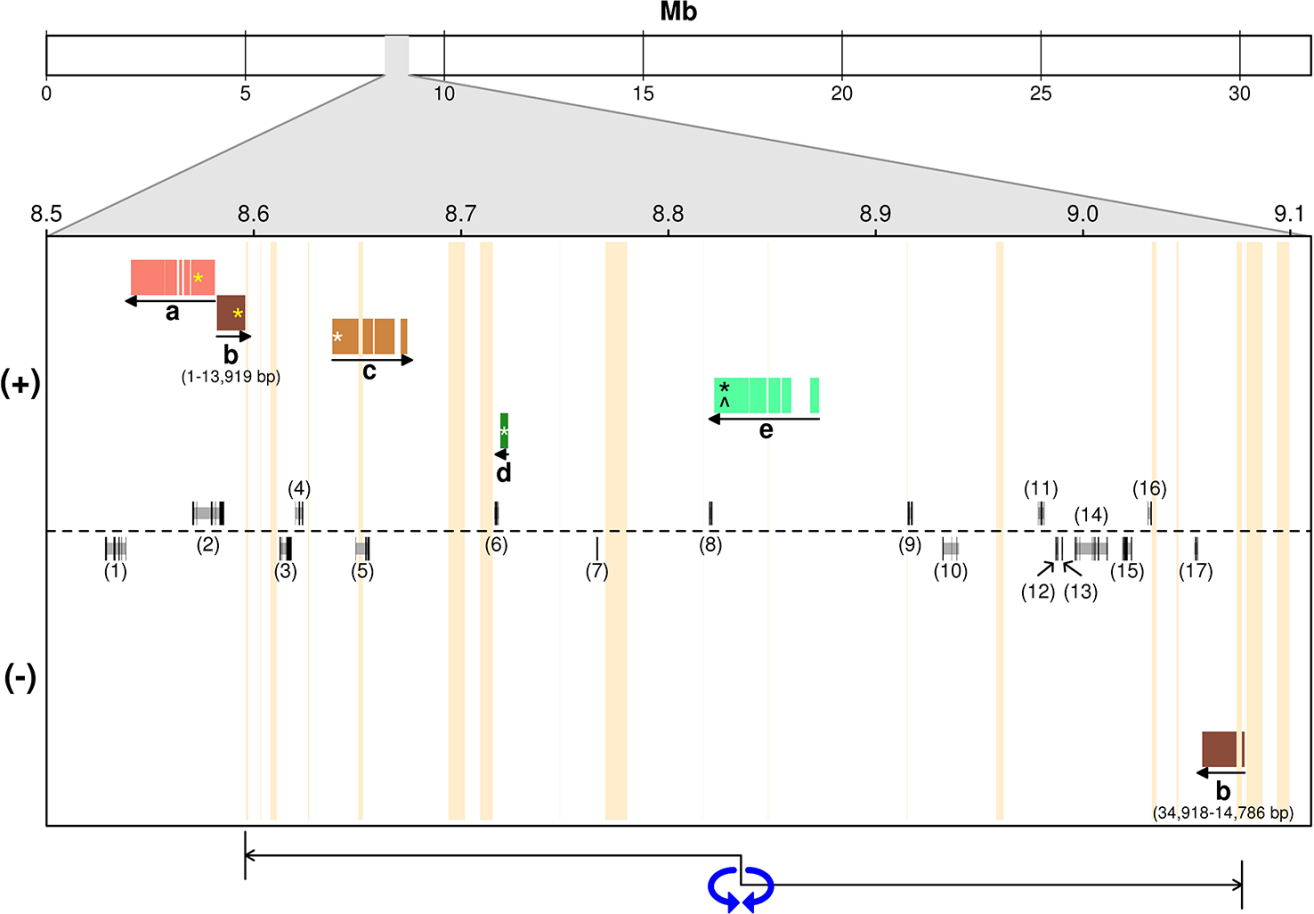
Alignment of ‘Leccino’ sequence scaffolds in the diallelic self-incompatibility (DSI) locus against the assembly of a wild olive genome (Unver et al., 2017). (+) and (−) indicate the strand of the wild olive genome sequence. Letters in the figure replace scaffolds IDs: a, scaffold_13712; b, scaffold_7600; c, scaffold_26872; d, scaffold_ 63515; e, scaffold_6030. The models above and below the dotted line indicate gene prediction in the wild olive genome on either strand. Numbers between parentheses identify the gene models described in **Supplementary Table S1**. Vertical orange bars indicate sequence gaps in wild olive assembly. Horizontal colored bars indicate the regions of alignment. Yellow asterisks on “a” and “b” indicate the position within each scaffold of the flanking restriction associated DNA (RAD) markers on one side the genetic locus. Asterisks indicate the position of RAD markers within each scaffold sequence that anchored that scaffold to the genetic map. White asterisks on “c” and “d” indicate the position within each scaffold of the flanking RAD markers on the other side the genetic locus. The black asterisk and the circumflex on “e” indicate the positions of co-segregating RAD and sequence-tagged site (STS) markers, respectively. The double-head arrow below indicates the region of order inconsistency between ‘Leccino’ map and the wild olive genome assembly.

The incompatibility locus was mapped as a Mendelian trait in the ‘Leccino’ map. The locus was located on LG 18 within an interval of 5.4 cM. Within this interval, the RAD markers on scaffold_6030 (sizing 42,057 bp) co-segregated with the trait in all 103 individuals of the progeny with a reliable incompatibility group assignment (**Figure 2**). The upper border of the locus was supported by a recombination event observed in one individual between the incompatibility group and two RAD markers located in scaffold_63515 (sizing 3,877 bp) and in scaffold_26872 (sizing 32,989 bp). The lower border of the genetic interval was supported by five crossing-over events observed in the progeny between the incompatibility group trait and a RAD marker in scaffold_7600 (sizing 34,918 bp) and by even more crossing-overs with a RAD marker in scaffold_13712 (sizing 37,403 bp). All these scaffolds of ‘Leccino’ aligned to a region between coordinates 8,500,000 and 9,100,000 of chromosome 18 in the genome assembly of a wild olive (Unver et al., 2017) (**Figure 3**).

### Alignment of Diallelic Self-Incompatibility Markers and Scaffolds With the Wild Olive Genome Assembly and Candidate Genes in the Region

The projection of ‘Leccino’ scaffolds containing the RAD flanking markers from either side of the DSI locus onto the assembly of chromosome 18 identified a seemingly short physical interval of a few dozen Kbs, comprised between scaffolds 13712 and 7600, on one side, and scaffold 26872 on the other side (**Figure 3**). The region around scaffolds 13712, 7600, 26872 in the wild olive assembly, corresponding to the chromosomal interval between 8.5 and 8.7 Mb, encodes five predicted proteins (**Supplementary Table S1**, IDs 1 to 5). However, the inconsistent physical position of scaffold_7600, which contains a RAD marker co-segregating with DSI and aligns outside of the interval defined by the flanking markers, and the split alignment of the initial 14 Kb of scaffold_7600 on one side of the locus and the remaining 12 Kb of scaffold_7600 on the opposite strand at the other side of the locus suggest that the physical interval might be substantially longer. A 450-kb inversion, indicated by a double-head arrow in **Figure 3**, or an assembly error in the wild olive genome between two flanking sequence gaps would reconcile the genetic marker order in the ‘Leccino’ map and the assembly of scaffold_7600 and would define a physical interval for the DSI locus in the wild olive assembly from coordinates 8,720,000 to 9,080,000, with inverted orientation (220 Kb-580 Kb relative coordinates in **Figure 3**). Under this hypothesis, the DSI region in the assembly of wild olive would encompass 12 additional predicted proteins, bringing the total number of candidate genes to 17 (**Supplementary Table S1**, IDs 6 to 17). RNA-Seq data also showed that other regions outside of the predicted gene models are transcribed in the physical interval of wild olive.

### Validation of Sequence-Tagged Site Markers Linked to the Incompatibility Group

**Figure 4:**
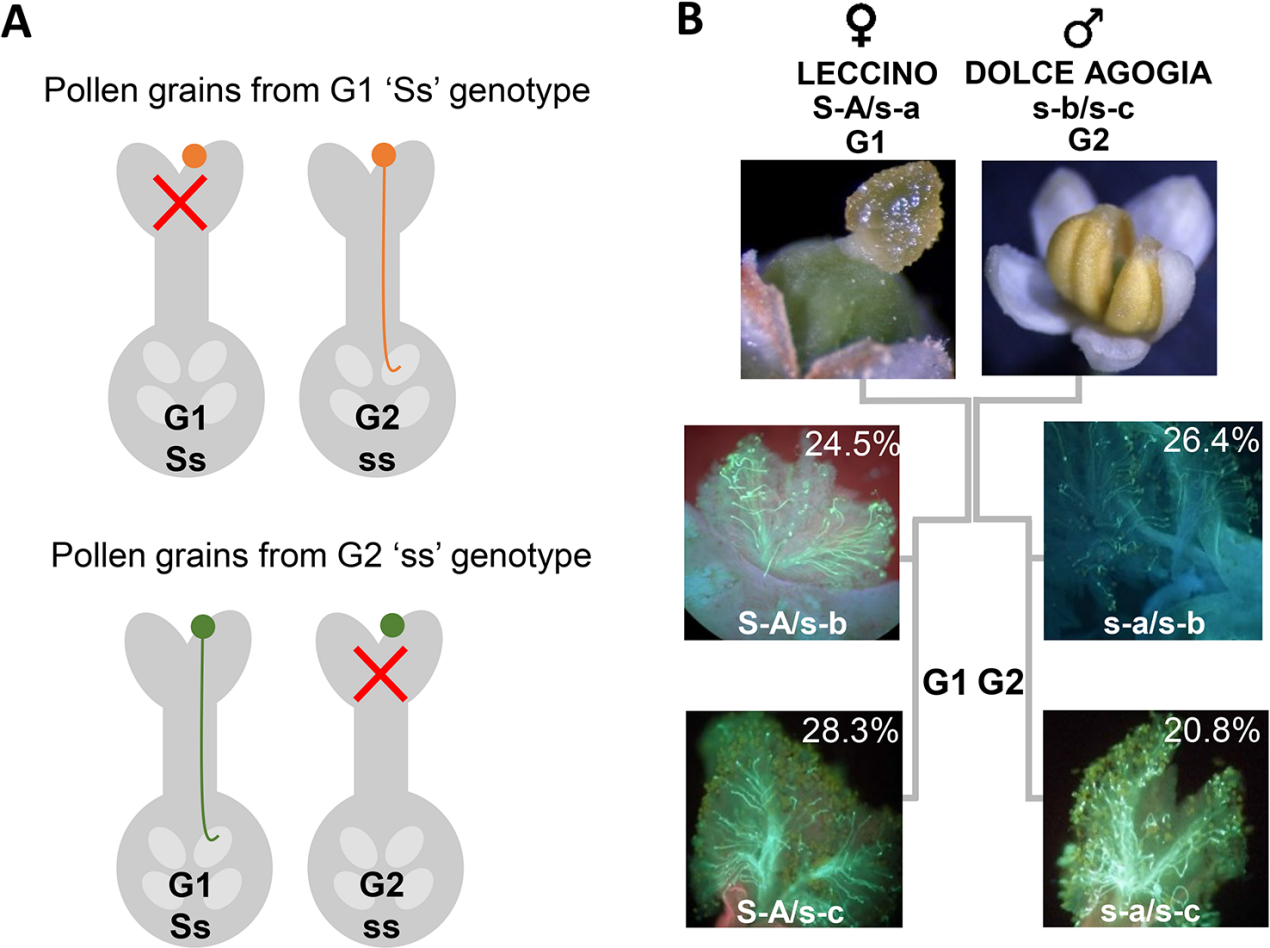
**(A)** Graphic illustration of the sporophytic diallelic self-incompatibility (DSI) system in olive. **(B)** Schematic representation of ‘Leccino’ (S-A/s-a) and ‘Dolce Agogia’ (s-b/s-c) allele combinations and segregation of four haplotypes in the Le×DA progenies.

We amplified and sequenced a PCR fragment (hereafter referred to as Oe-DSI-locus-fragment-A), spanning two SNPs on scaffold_6030 that showed co-segregation with RAD loci in ‘Leccino’ map. The sequencing results of 165 genotypes, which included cultivars and progenies, allowed to identify only two polymorphic sites at positions 63 and 283 bp of the Oe-DSI-locus-fragment-A, which define unambiguously the two groups of incompatibility (**Table 2** and **Supplementary Table S2**). In the parental cultivars Leccino and Dolce Agogia, the amplified fragment showed four alleles differentiated by eleven SNPs (**Table 3**). The inheritance of four haplotypes segregating from ‘Leccino’ (S-A/s-a) and ‘Dolce Agogia’ (s-b/s-c) and the linkage of the S-A haplotype with the genetic determinant of the G1 phenotype was confirmed by analyzing the Oe-DSI-locus-fragment-A in the Le×DA progeny (**Figure 4**). Combining the stigma test results with the genotypic data, the dominant allele S-A and three recessive ones, s-a, s-b, s-c, were identified.

**Table 2:** Incompatibility group phenotypes and corresponding haplotype combinations of Oe-DSI-locus-fragment-A observed in the analyzed olive cultivars.

Group of incompatibility	Haplotype combination	Diagnostic genotypes^a^^,^^b^
G1	S-B/-*	TT/-
	S-A/-*	TT/-
	S-A/s-a	TT/TC
	S-A/s-b	TG/TT
	S-A/s-c	TG/TT
G2	s-a/s-a	TT/CC
	s-a/s-b	TG/CT
	s-a/s-c	TG/CT
	s-b/s-b	GG/TT
	s-b/s-c	GG/TT
	s-c/s-c	GG/TT

a Genotype at position 63 bp of Oe-DSI-locus-fragment-A.

b Genotype at position 283 bp of Oe-DSI-locus-fragment-A.

*The second allele could be considered as null allele.

**Table 3:** Haplotypes of Oe-DSI-locus-fragment-A and position (base pair distance from the forward primer) of the polymorphisms [single-nucleotide polymorphisms (SNPs) and indels] identified. SNP combinations that identify uniquely each incompatibility group are indicated in bold.

Alleles	Accession N.	36	47	63	77	103	106	142	155	171	173	198	217	218	283
S-A	MN256463	T	G	**T**	T	T	TT	T	G	G	T	C	T	T	**T**
S-B	MN256465	T	G	**T**	C	C	–	T	G	G	T	C	C	-	**T**
s-a	MN256464	T	G	**T**	T	T	TT	C	A	A	C	T	T	T	**C**
s-b	MN256465	A	T	**G**	C	C	TT	T	G	G	T	C	T	T	**T**
s-c	MN256466	T	G	**G**	C	C	TT	T	G	G	T	C	T	T	**T**

When the same fragment was amplified in 57 olive cultivars with known incompatibility group assignment, an additional haplotype was found, the S-B allele, which represents a new dominant allele distinguishing G1 cultivars (**Supplementary Table S2**). The five haplotypes carried a total of 12 SNPs and 2 small indels (**Table 3**). In the 20 G1 cultivars, the dominant S-A allele was detected 13 times and S-B was present in apparent homozygous state in six out of seven cases and in one case with the haplotype s-b, detected by cloning. Both dominant alleles were found in combination with s-a, s-b, and s-c recessive alleles. The presence of only one dominant allele in some cultivars could be due to homozygosity or to the inability to amplify the second allele (null allele). Among the 37 G2 cultivars, the most frequent recessive haplotype was s-b, also showing a high percentage of homozygosity (**Supplementary Table S2**). A skewed geographical distribution was observed for some alleles between Italy and Spain, the two most represented countries in our data set, such the S-B allele, mainly present in Spanish cultivars, and s-c, only present within the Italian ones (**Supplementary Figure S1**).

## Discussion

Species with a long juvenile phase require many years to reach the first flowering and fruit setting (Purba et al., 2001; Flachowsky et al., 2011; Yang et al., 2016), delaying the generation of crossbreed, F2, and backcross populations and slowing down the process of genetic improvement. In these cases, the availability of genomic tools to assist the selection of new genotypes becomes mandatory in order to guide the choice of parental combinations, to select genotypes carrying the traits of interest, and to introgress specific alleles into new varieties. Markers linked to the traits under selection, identified either through genetic mapping approaches or by means of genome-wide association studies, represent the most powerful tools for breeding in woody perennial crops, offering new opportunities to develop early selection strategies and new ways to integrate variation from different sources (Varshney et al., 2005; Montanari et al., 2013; Bink et al., 2014; Kole et al., 2015; Muranty et al., 2015).

The use of high-throughput sequencing technologies is speeding up the genotyping of mapping populations, providing an unprecedented high number of markers that allows the construction of dense genetic maps (Kujur et al., 2015; Liu et al., 2017). In this work, we have used ddRAD sequencing for genotyping 7,006 and 9,737 segregating sites in a biparental population, which largely exceeded the number of genetic bins that can be resolved in a progeny of 229 individuals. Olive tree is known to be a highly heterozygous species with very high levels of nucleotide diversity observed even in cultivated varieties (Gros-Balthazard et al., 2019). Here we show that the species is characterized also by very high levels of recombination as attested by the lengths of the two parental genetic maps. We observed a significant difference in the length between the male Dolce Agogia (3,538 cM) and the female Leccino (5,680 cM) maps. Heterochiasmy, i.e., the presence of different crossover frequencies in male and female meiosis, has frequently been observed in plants, without a fixed trend of higher frequency in male or female meiosis (Lenormand and Dutheil, 2005). In *Arabidopsis*, for example, a dramatically higher crossing over rate (575 cM *vs.* 332 cM) is observed in male than in female meiosis (Giraut et al., 2011). Theory would predict that haploid selection determines heterochiasmy, with the sex experiencing more intense selection during the haploid phase showing lower recombination (Lenormand and Dutheil, 2005). In a highly heterozygous and obligately outcrossing species such as olive, we can expect stronger selection among male gametophytes that could explain the observed difference in genetic map length. An alternative explanation could be provided by a high frequency of chromosomal inversions or other chromosomal rearrangements that suppress recombination. The identification of two large inversions for which the two parental varieties are homozygous for alternative alleles makes us believe that in such a highly heterozygous species, each individual accession may be heterozygous for a much larger set of inversions. As ‘Dolce Agogia’ appears to be more heterozygous than ‘Leccino,’ the lower recombination frequency observed could be explained by a higher frequency of heterozygous chromosomal rearrangements that suppress recombination.

In this case, the generation of markers did not represent a limiting factor for trait mapping, whose resolution was rather limited by the size of the progeny subject to phenotyping. Phenotyping of reproductive traits requires adult plants and, in the case of DSI group assignment, is also labor intensive. We mapped the DSI locus to a 5.4 cM genetic interval, which is estimated to correspond to a physical distance of approximately 300 Kb in a chromosome-scale assembly of a wild olive genome (Unver et al., 2017). Gene prediction and gene annotation in the wild olive assembly did not provide any obvious functional candidate, except for transcription factors that might be involved in gynoecium development regulation. Two candidate genes encode proteins putatively related to flower development: the ortholog of *Arabidopsis* STYLISH 1 encoding a binding protein with nuclear localization that promotes formation of stylar and stigmatic tissues and proliferation of stylar xylem (Gomariz-Fernández et al., 2017; Min et al., 2019), and FAR1 related sequence 5, which is expressed in hypocotyls, inflorescences stems and flowers (Li et al., 2017; Ma and Li, 2018). However, the prediction of proteins with uncharacterized function in the same region, the presence of non-annotated transcribed regions, the expected intraspecific presence/absence variation between genomes, make it necessary to proceed with the complete assembly of the two ‘Leccino’ haplotypes across the entirety of the DSI locus.

While we confirmed the monogenic nature of the DSI system in olive, as postulated by Saumitou-Laprade et al. (2017a), five haplotypes were identified using a sequence-tagged site (STS) marker in the locus and, *via* linkage mapping and association mapping, we demonstrated that two of them are in phase with the dominant genetic determinant of the G1 incompatibility group. The STS haplotypes were linked to the DSI genetic determinant, but they do not correspond to the DSI alleles. It was noteworthy that the cumulative frequency of the STS haplotypes in phase with the dominant S allele was roughly half the cumulative frequency of haplotypes in phase with the s allele, although the accessions analyzed in this paper are not a natural population. It is possible to speculate that this condition may reflect a balancing selection for maintaining G1 and G2 genotypes in a cultivated population at frequencies that maximize pollination rate.

In the set of cultivars analyzed in this paper, we found only two dominant S-A and S-B alleles that confer the G1 phenotype and no cultivar was observed carrying both dominant alleles. It was also noteworthy that some haplotypes were more frequent in groups of cultivars typical of specific geographic locations. For instance, the allele s-c showed 18% frequency in the sample and was almost exclusively present in varieties of Central Italy, leading to an excess of homozygous genotypes in that geographic area, whereas the S-B haplotype was mostly present in cultivars from the Iberian Peninsula. Variation of haplotype frequencies at DSI-linked markers in geographically distant populations may simply reflect genetic drift or may indicate adaptive evolution changing the frequency of DSI alleles. Further analyses will help to clarify these issues and to identify additional untapped variation at the DSI locus.

From an evolutionary point of view, the maintenance of a homomorphic DSI in a hermaphrodite species like *O. europaea* is unexpected and remains to be explained. Indeed, the SI systems are susceptible to rapid invasion by new self-incompatibility alleles, which should experience a strong negative frequency-dependent advantage. In *Oleaceae* species in which DSI was identified, such invasion was not observed (Saumitou-Laprade et al., 2010; Vernet et al., 2016; Saumitou-Laprade et al., 2017a; Saumitou-Laprade et al., 2017b; Saumitou-Laprade et al., 2018). The homomorphic DSI system seems to “resist” and this is an intriguing finding (Barrett, 2019). In the absence of obvious evolutionary constraints that could prevent the selection of new SI specificities, the molecular constraint appears to be the best candidate. Therefore, the molecular characterization of the S locus region in *Olea* and its comparison with other *Oleaceae* species are of strong interest. The present work is opening the way and should contribute efficiently to solve the evolutionary paradox of the stable homomorphic DSI in *Oleaceae*.

The STS marker identified in the present work represents a new tool for large-scale screening of thousands of olive accessions from their traditional areas of cultivation across the Mediterranean shores and from new growing areas and will allow to determine their incompatibility group. In addition, it represents a starting point for the identification of the genetic determinant of such a peculiar incompatibility system. A comprehensive understanding of the genetic control of DSI can offer great opportunities to characterize cultivars for their incompatibility group, increase the olive production and guide the orchard plantation design with optimal spatial distribution of inter-compatible varieties.

## Conclusions

This work provides markers for a fast and reliable genotyping of olive cultivars for their incompatibility group, offering great opportunities to rapidly screen and identify inter-compatible varieties, planning inter-varietal crosses, and reducing the time for seedling selection. It also represents the initial effort for the identification of the genetic determinants of incompatibility, a starting point for understanding the molecular mechanisms underlying the DSI system in olive.

The genetic maps of ‘Leccino’ and ‘Dolce Agogia’ will also serve to identify Mendelian loci or QTLs responsible for other important traits that segregate in the Le×DA progeny. Information yet to be gained on the number and location of the genetic determinants of those traits, along with the DSI-linked ddRAD and STS markers developed in this paper, may pave the way to the application of genomics-assisted breeding in olive.

## Data Availability Statement

Raw reads of ddRAD-Seq have been deposited in Short Read Archive under the BioProject number PRJNA594490. The matrix of genotypic calls has been deposited in the figshare repository with the DOI 10.6084/m9.figshare.11352068.

## Author Contributions

RM, PS-L, PV, MM and LB conceived the study. SP, RM, NC and LB provided the plant material. RM, SM, MR, PS-L, PV and FA performed the stigma tests. RM, NC, VP, MR, AF, GM, SS, DS, GDG and MM performed the molecular, sequencing and bioinformatic analyses. FB, LB, RM, SM and VP wrote the first draft of the manuscript. PS-L, PV, DS, GDG and MM contributed to the writing and revision of the manuscript. All the authors agreed on the final version of this work.

## Funding

The research was supported by the European Union’s Horizon 2020 Research and Innovation Program Marie Sklodowska-Curie - Before Project (Grant Agreement No 645595), by the EU projects “OLIVE4CLIMATE – LIFE” (LIFE15 CCM/IT/000141) and by the Rural Development Program of Umbria Region, 2014-2020 – Measure 16.2.1, INNO.V.O. - Development of alternative varieties to face the new challenges of olive growing, SIAN n. 84250258245.

## Conflict of Interest

The authors declared that the research was conducted in the absence of any commercial or financial relationships that could be construed as a potential conflict of interest.

## Supplementary Material

The Supplementary Material for this article can be found online at: https://www.frontiersin.org/articles/10.3389/fpls.2019.01760/full#supplementary-material

Supplementary Figure S1 Geographical distribution of DSI alleles in the Italian and Spanish analyzed varieties.
