# Supplementary figures and images for: Genetic Mapping of the Incompatibility Locus in Olive and Development of a Linked Sequence-Tagged Site Marker
Source: Front Plant Sci. 2020 Jan 28;10:1760. doi: 10.3389/fpls.2019.01760 (PMC7025539; doi:10.3389/fpls.2019.01760)

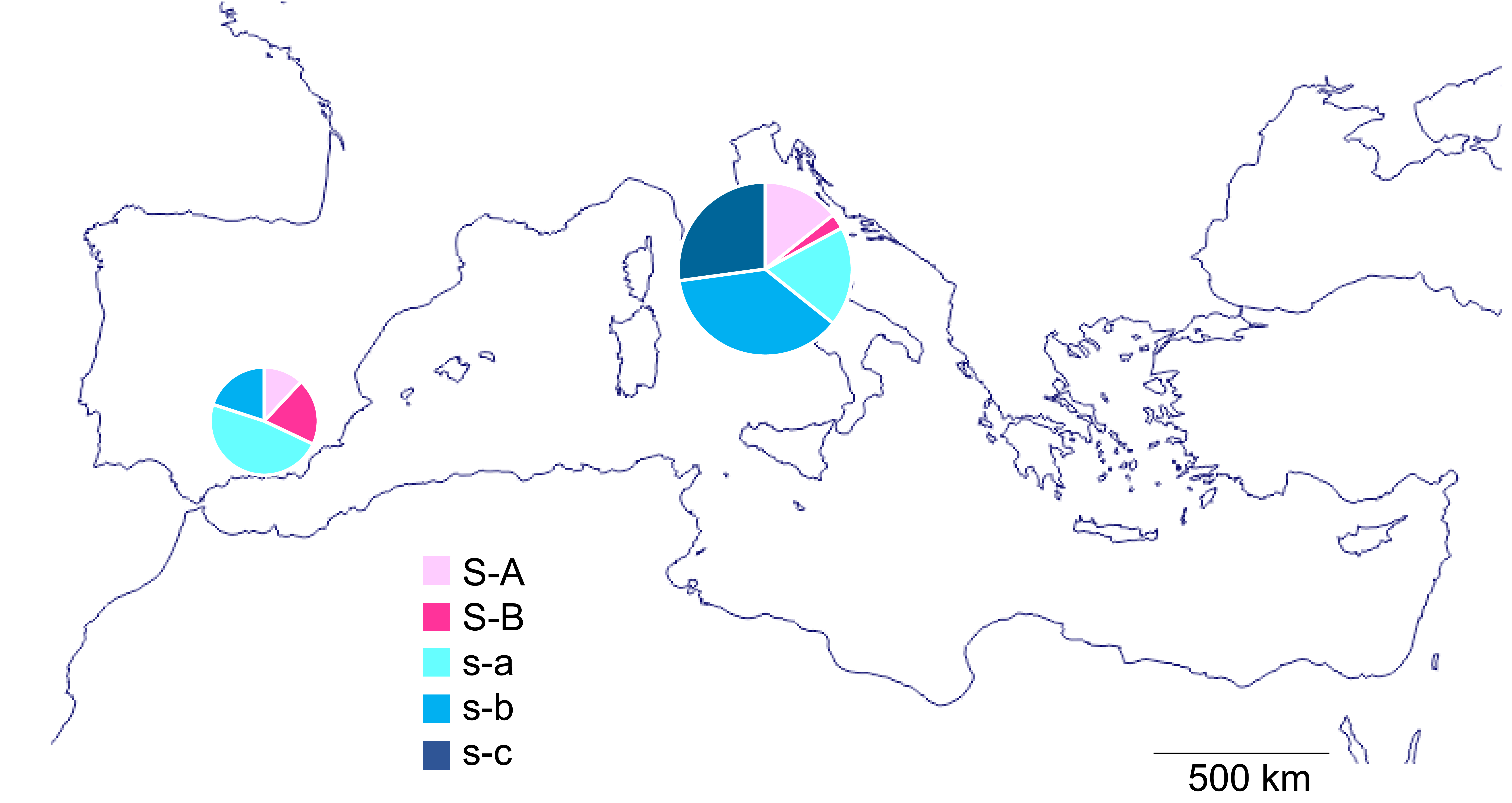

Supplement: Supplementary Figure S1 — Geographical distribution of DSI alleles in the Italian and Spanish analyzed varieties. [file Image_1.jpeg]
