# Supplementary material for: Genetic Mapping of the Incompatibility Locus in Olive and Development of a Linked Sequence-Tagged Site Marker
Source: Front Plant Sci. 2020 Jan 28;10:1760. doi: 10.3389/fpls.2019.01760 (PMC7025539; doi:10.3389/fpls.2019.01760)
Supplement: Supplementary file 2 [file Table_1.docx]

**Supplementary Table S1.** Annotation of the predicted gene models in the chromosome interval of wild olive corresponding to the DSI locus in ‘Leccino’.

| **ID in Fig.3** | **Gene ID** | **Location** | **Coding strand** | **Functional annotation** |
| --- | --- | --- | --- | --- |
| 1 | LOC111367381 | chr18:8,528,227..8,538,408 | - | Chaperone-like protein of POR1 |
| 2 | LOC111367383 | chr18:8,570,307..8,585,694 | + | Zinc finger CCCH domain-containing protein 44 |
| 3 | LOC111367384 | chr18:8,612,415..8,618,260 | - | Uncharacterized protein |
| 4 | LOC111366697 | chr18:8,620,048..8,623,927 | + | FAR1-related sequence 5 |
| 5 | LOC111367385 | chr18:8,649,039..8,655,863 | - | SHI related sequence 1 (STYLISH 1) |
| 6 | LOC111366698 | chr18:8,715,988..8,716,048 | + | 61-bp pseudogene, probable methyltransferase PMT27 |
| 7 | LOC111367386 | chr18:8,765,216..8,766,001 | - | Uncharacterized protein |
| 8 | LOC111367387 | chr18:8,819,582..8,821,267 | + | GATA transcription factor 5 |
| 9 | LOC111367389 | chr18:8,915,356..8,917,792 | + | Cytochrome P450 84A1 |
| 10 | LOC111367390 | chr18:8,932,208..8,940,148 | - | Uncharacterized protein |
| 11 | LOC111366699 | chr18:8,978,278..8,981,237 | + | Uncharacterized protein |
| 12 | LOC111366700 | chr18:8,986,696..8,988,082 | - | Uncharacterized protein |
| 13 | LOC111367391 | chr18:8,989,633..8,990,335 | - | ncRNA |
| 14 | LOC111367392 | chr18:8,995,947..9,012,004 | - | Transmembrane and coiled-coil domain-containing protein 4 |
| 15 | LOC111367393 | chr18:9,019,094..9,023,583 | - | Zinc finger CCCH domain-containing protein 44, partial |
| 16 | LOC111366701 | chr18:9,031,220..9,033,134 | + | 60S ribosomal protein L18-2, partial |
| 17 | LOC111366702 | chr18:9,053,580..9,055,405 | - | Uncharacterized protein |

**Supplementary Table S2.** List of olive genotypes (cultivars and LexDA offsprings) phenotyped by a stigma test and genotyped by re-sequencing the alleles identified within the incompatibility locus in the map of cv. Leccino. The group of incompatibility, the haplotype combinations and the diagnostic SNP combinations (at 63 and 283 bp position, respectively) are reported for all samples.

| **Cultivars** | **Country of diffusion** | **Group of incompatibility** | **Haplotype combination** | **Diagnostic SNPs** |
| --- | --- | --- | --- | --- |
| Gordal Sevillana | Spain | G1 | S-A/s-a | TT/TC |
| Leccino | Italy | G1 | S-A/s-a | TT/TC |
| Piantone di Falerone | Italy | G1 | S-A/s-a | TT/TC |
| Arbequina | Spain | G1 | S-A/s-b | TG/TT |
| Cornicabra | Spain | G1 | S-A/s-b | TG/TT |
| Dritta di Muscufo | Italy | G1 | S-A/s-b | TG/TT |
| Gentile di Montone | Italy | G1 | S-A/s-b | TG/TT |
| Gentile Grande | Italy | G1 | S-A/s-b | TG/TT |
| Sirole | Italy | G1 | S-A/s-b | TG/TT |
| Frantoio | Italy | G1 | S-A/s-c | TG/TT |
| Moraiolo | Italy | G1 | S-A/s-c | TG/TT |
| Vera Umbra | Italy | G1 | S-A/s-c | TG/TT |
| Moresca | Italy | G1 | S-B/s-b | TG/TT |
| Cornezuelo de Jaen | Spain | G1 | S-B/-* | TT/- |
| Hojiblanca | Spain | G1 | S-B/-* | TT/- |
| Lechin de Sevilla | Spain | G1 | S-B/-* | TT/- |
| Orbetana | Italy | G1 | S-B/-* | TT/- |
| Sevillenca | Spain | G1 | S-B/-* | TT/- |
| Verdial de Badajoz | Spain | G1 | S-B/-* | TT/- |
| Leccio del Corno | Italy | G1 | S-A/-* | TT/- |
| Bottone di Gallo | Italy | G2 | s-a/s-a | TT/CC |
| Machorron | Spain | G2 | s-a/s-a | TT/CC |
| Manzanilla Cacerena | Spain | G2 | s-a/s-a | TT/CC |
| Mollar de Cieza | Spain | G2 | s-a/s-a | TT/CC |
| Picual | Spain | G2 | s-a/s-a | TT/CC |
| Picudo | Spain | G2 | s-a/s-a | TT/CC |
| Arauco | Argentina | G2 | s-a/s-b | TG/CT |
| Borgiona | Italy | G2 | s-a/s-b | TG/CT |
| Carolea | Italy | G2 | s-a/s-b | TG/CT |
| Itrana | Italy | G2 | s-a/s-b | TG/CT |
| Manzanilla de Sevilla | Spain | G2 | s-a/s-b | TG/CT |
| Mastoidis | Greece | G2 | s-a/s-b | TG/CT |
| Nocellara del Belice | Italy | G2 | s-a/s-b | TG/CT |
| Passalunara | Italy | G2 | s-a/s-b | TG/CT |
| Piangente | Italy | G2 | s-a/s-b | TG/CT |
| Picholine Marocaine | Morocco | G2 | s-a/s-b | TG/CT |
| Maurino | Italy | G2 | s-a/s-c | TG/CT |
| Nostrale di Rigali | Italy | G2 | s-a/s-c | TG/CT |
| Semidana | Italy | G2 | s-a/s-c | TG/CT |
| Verdale | France | G2 | s-a/s-c | TG/CT |
| Arbosana | Spain | G2 | s-b/s-b | GG/TT |
| Capolga | Italy | G2 | s-b/s-b | GG/TT |
| Fecciaro | Italy | G2 | s-b/s-b | GG/TT |
| Gnacolo | Italy | G2 | s-b/s-b | GG/TT |
| Grappuda | Italy | G2 | s-b/s-b | GG/TT |
| Koroneiki | Greece | G2 | s-b/s-b | GG/TT |
| Morrut | France | G2 | s-b/s-b | GG/TT |
| Raio | Italy | G2 | s-b/s-b | GG/TT |
| Sant'Emiliano | Italy | G2 | s-b/s-b | GG/TT |
| Tombareddu | Italy | G2 | s-b/s-b | GG/TT |
| Dolce Agogia | Italy | G2 | s-b/s-c | GG/TT |
| Bosana | Italy | G2 | s-c/s-c | GG/TT |
| Canino | Italy | G2 | s-c/s-c | GG/TT |
| Coratina | Italy | G2 | s-c/s-c | GG/TT |
| Gentile di Chieti | Italy | G2 | s-c/s-c | GG/TT |
| Mignola | Italy | G2 | s-c/s-c | GG/TT |
| Pendolino | Italy | G2 | s-c/s-c | GG/TT |
| **Le×DA progeny** |  | **Group of incompatibility** | **Haplotype combination** | **Diagnostic SNPs** |
| 202 |  | G1 | S-A/s-b | TG/TT |
| 205 |  | G1 | S-A/s-b | TG/TT |
| 210 |  | G1 | S-A/s-b | TG/TT |
| 211 |  | G1 | S-A/s-b | TG/TT |
| 213 |  | G1 | S-A/s-b | TG/TT |
| 214 |  | G1 | S-A/s-b | TG/TT |
| 238 |  | G1 | S-A/s-b | TG/TT |
| 245 |  | G1 | S-A/s-b | TG/TT |
| 247 |  | G1 | S-A/s-b | TG/TT |
| 249 |  | G1 | S-A/s-b | TG/TT |
| 252 |  | G1 | S-A/s-b | TG/TT |
| 268 |  | G1 | S-A/s-b | TG/TT |
| 272 |  | G1 | S-A/s-b | TG/TT |
| 276 |  | G1 | S-A/s-b | TG/TT |
| 284 |  | G1 | S-A/s-b | TG/TT |
| 285 |  | G1 | S-A/s-b | TG/TT |
| 300 |  | G1 | S-A/s-b | TG/TT |
| 312 |  | G1 | S-A/s-b | TG/TT |
| SP10 |  | G1 | S-A/s-b | TG/TT |
| SP15 |  | G1 | S-A/s-b | TG/TT |
| SP26 |  | G1 | S-A/s-b | TG/TT |
| SP33 |  | G1 | S-A/s-b | TG/TT |
| SP73 |  | G1 | S-A/s-b | TG/TT |
| SP75 |  | G1 | S-A/s-b | TG/TT |
| SP89 |  | G1 | S-A/s-b | TG/TT |
| SP90 |  | G1 | S-A/s-b | TG/TT |
| 201 |  | G1 | S-A/s-c | TG/TT |
| 206 |  | G1 | S-A/s-c | TG/TT |
| 230 |  | G1 | S-A/s-c | TG/TT |
| 237 |  | G1 | S-A/s-c | TG/TT |
| 246 |  | G1 | S-A/s-c | TG/TT |
| 257 |  | G1 | S-A/s-c | TG/TT |
| 259 |  | G1 | S-A/s-c | TG/TT |
| 267 |  | G1 | S-A/s-c | TG/TT |
| 270 |  | G1 | S-A/s-c | TG/TT |
| 273 |  | G1 | S-A/s-c | TG/TT |
| 279 |  | G1 | S-A/s-c | TG/TT |
| 286 |  | G1 | S-A/s-c | TG/TT |
| 289 |  | G1 | S-A/s-c | TG/TT |
| 291 |  | G1 | S-A/s-c | TG/TT |
| 297 |  | G1 | S-A/s-c | TG/TT |
| 298 |  | G1 | S-A/s-c | TG/TT |
| 303 |  | G1 | S-A/s-c | TG/TT |
| 306 |  | G1 | S-A/s-c | TG/TT |
| 307 |  | G1 | S-A/s-c | TG/TT |
| 308 |  | G1 | S-A/s-c | TG/TT |
| SP16 |  | G1 | S-A/s-c | TG/TT |
| SP17 |  | G1 | S-A/s-c | TG/TT |
| SP21 |  | G1 | S-A/s-c | TG/TT |
| SP63 |  | G1 | S-A/s-c | TG/TT |
| SP70 |  | G1 | S-A/s-c | TG/TT |
| SP74 |  | G1 | S-A/s-c | TG/TT |
| SP81 |  | G1 | S-A/s-c | TG/TT |
| SP83 |  | G1 | S-A/s-c | TG/TT |
| SP85 |  | G1 | S-A/s-c | TG/TT |
| SP93 |  | G1 | S-A/s-c | TG/TT |
| 212 |  | G2 | s-a/s-b | TG/TC |
| 218 |  | G2 | s-a/s-b | TG/TC |
| 222 |  | G2 | s-a/s-b | TG/TC |
| 228 |  | G2 | s-a/s-b | TG/TC |
| 232 |  | G2 | s-a/s-b | TG/TC |
| 234 |  | G2 | s-a/s-b | TG/TC |
| 236 |  | G2 | s-a/s-b | TG/TC |
| 242 |  | G2 | s-a/s-b | TG/TC |
| 251 |  | G2 | s-a/s-b | TG/TC |
| 253 |  | G2 | s-a/s-b | TG/TC |
| 258 |  | G2 | s-a/s-b | TG/TC |
| 262 |  | G2 | s-a/s-b | TG/TC |
| 263 |  | G2 | s-a/s-b | TG/TC |
| 266 |  | G2 | s-a/s-b | TG/TC |
| 277 |  | G2 | s-a/s-b | TG/TC |
| 290 |  | G2 | s-a/s-b | TG/TC |
| 296 |  | G2 | s-a/s-b | TG/TC |
| 301 |  | G2 | s-a/s-b | TG/TC |
| SP28 |  | G2 | s-a/s-b | TG/TC |
| SP29 |  | G2 | s-a/s-b | TG/TC |
| SP30 |  | G2 | s-a/s-b | TG/TC |
| SP39 |  | G2 | s-a/s-b | TG/TC |
| SP53 |  | G2 | s-a/s-b | TG/TC |
| SP55 |  | G2 | s-a/s-b | TG/TC |
| SP56 |  | G2 | s-a/s-b | TG/TC |
| SP57 |  | G2 | s-a/s-b | TG/TC |
| SP78 |  | G2 | s-a/s-b | TG/TC |
| SP92 |  | G2 | s-a/s-b | TG/TC |
| 204 |  | G2 | s-a/s-c | TG/TC |
| 207 |  | G2 | s-a/s-c | TG/TC |
| 217 |  | G2 | s-a/s-c | TG/TC |
| 226 |  | G2 | s-a/s-c | TG/TC |
| 229 |  | G2 | s-a/s-c | TG/TC |
| 239 |  | G2 | s-a/s-c | TG/TC |
| 244 |  | G2 | s-a/s-c | TG/TC |
| 255 |  | G2 | s-a/s-c | TG/TC |
| 260 |  | G2 | s-a/s-c | TG/TC |
| 261 |  | G2 | s-a/s-c | TG/TC |
| 264 |  | G2 | s-a/s-c | TG/TC |
| 282 |  | G2 | s-a/s-c | TG/TC |
| 287 |  | G2 | s-a/s-c | TG/TC |
| 294 |  | G2 | s-a/s-c | TG/TC |
| 299 |  | G2 | s-a/s-c | TG/TC |
| 302 |  | G2 | s-a/s-c | TG/TC |
| 311 |  | G2 | s-a/s-c | TG/TC |
| 313 |  | G2 | s-a/s-c | TG/TC |
| 315 |  | G2 | s-a/s-c | TG/TC |
| SP35 |  | G2 | s-a/s-c | TG/TC |
| SP41 |  | G2 | s-a/s-c | TG/TC |
| SP51 |  | G2 | s-a/s-c | TG/TC |

*The second allele has not been reported because of the uncertain homozygous status of these genotypes. Null alleles could be considered.
